# Hitchhiking Robots: A Collaborative Approach for Efficient Multi-Robot Navigation in Indoor Environments

**DOI:** 10.3390/s17081878

**Published:** 2017-08-15

**Authors:** Abhijeet Ravankar, Ankit A. Ravankar, Yukinori Kobayashi, Takanori Emaru

**Affiliations:** Lab of Robotics and Dynamics, Faculty of Engineering, Hokkaido University, Sapporo 060-8628, Japan; ankit@eng.hokudai.ac.jp (A.A.R.); kobay@eng.hokudai.ac.jp (Y.K.); emaru@eng.hokudai.ac.jp (T.E.)

**Keywords:** multi-robot navigation, multi-robot cooperation, indoor robot systems

## Abstract

Hitchhiking is a means of transportation gained by asking other people for a (free) ride. We developed a multi-robot system which is the first of its kind to incorporate hitchhiking in robotics, and discuss its advantages. Our method allows the hitchhiker robot to skip redundant computations in navigation like path planning, localization, obstacle avoidance, and map update by completely relying on the driver robot. This allows the hitchhiker robot, which performs only visual servoing, to save computation while navigating on the common path with the driver robot. The driver robot, in the proposed system performs all the heavy computations in navigation and updates the hitchhiker about the current localized positions and new obstacle positions in the map. The proposed system is robust to recover from ‘driver-lost’ scenario which occurs due to visual servoing failure. We demonstrate robot hitchhiking in real environments considering factors like service-time and task priority with different start and goal configurations of the driver and hitchhiker robots. We also discuss the admissible characteristics of the hitchhiker, when hitchhiking should be allowed and when not, through experimental results.

## 1. Introduction

Hitchhiking is a social and symbiotic behavior with many potential advantages for the hitchhiker. The hitchhiker: (a) does not need to do path planning; (b) does not need to localize himself while navigation; (c) does not need to worry about control sequences (ex. car steering); (d) does not need to do obstacle avoidance, and (e) saves energy and money. Now consider a multi-robot system deployed at a large infrastructure like a warehouse for object delivery, cleaning, patrolling, and other tasks. Each robot of the multi-robot system needs to navigate from one location to other to perform its task. The paths of the robots often overlap completely or partially. Moreover, each robot is equipped with its own path planning unit, localization unit, obstacle avoidance unit, and SLAM (Simultaneous Localization and Mapping) [1,2] unit. In the context of hitchhiking, following questions naturally arise: Why should two robots with completely or partially overlapping paths separately perform their own path planning, collision avoidance, localization, and new obstacle update? Can’t one robot just hitchhike another robot going to the (nearly) same location, and skip most of the redundant operations?

Although there exists a plethora of literature on leader-follower multi-robot systems, the presented work is the first to address this question and demonstrate and discuss the feasibility and advantages of hitchhiking in multi-robot systems in various scenarios, to the best of our survey. Multi-robot systems and especially leader-follower robot systems have been presented earlier, however to realize different objectives. A multi-robot leader-follower system using two tractor robots has been proposed in [3,4] to improve efficiency in agricultural tasks in which the focus is on cooperation and coordination to execute a turn without collision. Many works have been proposed in collective localization [5] and mapping [6,7,8]. However, in all these works, the robots are independent entities and the emphasis is on using sensor data from different robots to collectively build maps and localize in the environment. An interesting technique to arrange robot rendezvous, i.e., pairing of two robots in an unknown environment is proposed [9] for collaborative exploration. Similarly, multi-robot navigation has been proposed [10,11,12] with focus on collision avoidance and area coverage. A semi-autonomous teleoperation system is proposed in [13] in which the follower robot satisfies several constraints while tracking the leader robot. The emphasis is on making the follower robot semi-autonomous when teleoperating the follower is difficult in cluttered environments. A similar scheme is presented in [14] for efficient teleoperational control of master-slave robots.

All the aforementioned works make important contributions but with different goals. To the best of our knowledge, none of the research has discussed how a follower robot can skip path-planning, obstacle avoidance, localization, and map update by completely relying on the leader robot for navigation towards a (nearly) common goal location. We experimentally show the feasibility of such ‘hitchhking’ with experimental results in real environment. We particularly focus on strategies in which the hitchhiker does not lose any information (like new obstacles) in the environment by shutting down some of its modules, and is able to recover in case of failure in following the driver robot.

## 2. Hitchhiking in Robots

The following terminologies are used throughout the paper: (a) Driver: is the leader robot in the leader-follower scene. Driver does all the path planning, obstacle avoidance, map update, and assists the follower robot in the hitchhiking process; (b) Hitchhiker: is the robot which follows the driver through visual servoing, and shuts down other modules.

### 2.1. Hitchhiking Mechanism

Hitchhiking is achieved through visual servoing. A complete description of visual servoing is beyond the scope of the main idea of the proposed work. However, a brief description is provided.

Visual servoing [15], also known as vision-based robot control is a technique which uses feedback information extracted from a vision sensor (like camera) to control the motion of a robot. Visual Servoing control techniques are broadly classified into the following types [16]: (a) Image-based Visual Servoing (IBVS) [17], which calculates the error between desired features (ex. lines, points) in images without estimating the pose of the target and has problems with large rotational motions; (b) Position/pose-based Visual Servoing (PBVS) [18], a model-based technique which estimates also the pose of the target with respect to the camera using the extracted image features enabling servoing in 3D; (c) Hybrid approach based Visual Servoing [19] which uses a combination of the 2D and 3D servoing.

A set of visual features *m* are extracted from a set of visual measurements x(t) which comprises of coordinates of points of interest, i.e.,

(1) $$\begin{array}{c} m=m(x(t)), \end{array}$$

which allows the required degrees of freedom [20]. For correct realization, a controller is designed such that the features *m* reach a desired value $m^{*}$, such that the error vector ${m-m}^{*}$ is zero. In vision based control, the objective is to minimize an error e(t), where,

(2) $$\begin{array}{c} e(t)={m-m}^{*}. \end{array}$$

The required trajectory $m^{*}\left(t\right)$ is generated and details can be found in [16,21].

**Figure 1:**
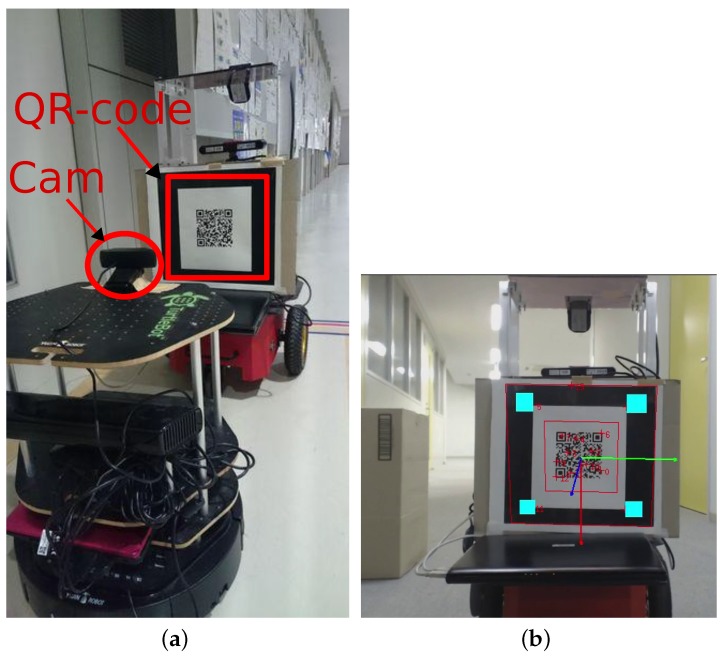
QR code and camera system for coupling. (**a**) Robot with fixed QR code and camera for visual servoing; (**b**) An example of pose estimation from QR-code in visual servoing.

Visual servoing is generally implemented through an artificial marker and a camera. In our implementation, each robot has a QR-code which is fixed on the back side of the robot. Each robot also has a forward facing camera. The QR-code and camera setup is shown in Figure 1a.

### 2.2. Four Steps of Hitchhiking

Each robot of the multi-robot system is equipped with path planning, localization and mapping, obstacle avoidance, communication, and other necessary modules. Each robot is provided with a unique ID ($R_{\mathrm{id}}$). They also have respective start ($S_{\mathrm{loc}}$) and goal location ($G_{\mathrm{loc}}$), task priority ($T_{p}$), and profile ($P_{\mathrm{id}}$). A profile comprises of the specifications of the robot, i.e., the type of sensors attached to the robot, accuracy of the odometers and sensors, and robustness of the SLAM module. In actual implementation, a numerical value is manually assigned against each attribute (the better the attribute, higher the value). A profile provides logical parameters to compare the specifications of the two robots.

**Figure 2:**
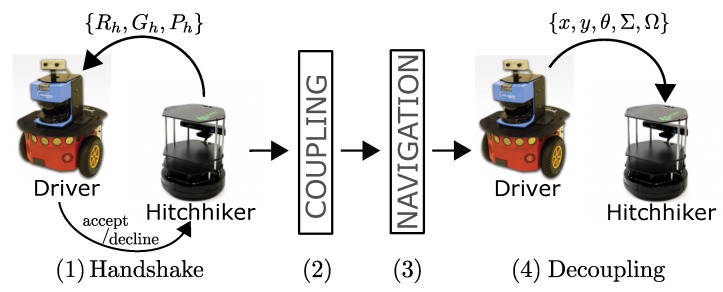
Four steps of hitchhiking: (1) Handshake, (2) Coupling, (3) Navigation, and (4) Decoupling.

**Figure 3:**
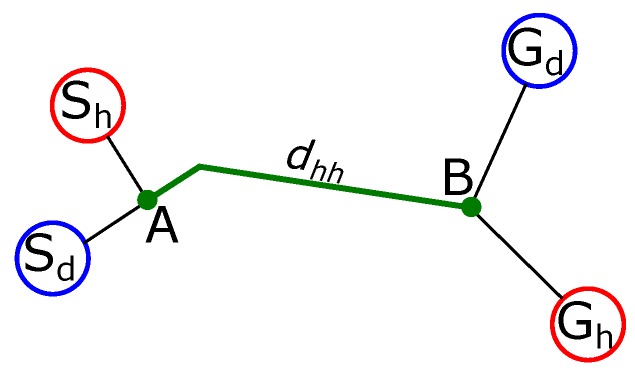
Hitchhking is allowed if the common path $d_{hh}$ is longer than a threshold distance ($T_{dhh}$). Point A is the handshake and coupling location, while point B is the decoupling location.

Hitchhiking is carried out in the four steps which are graphically shown in Figure 2 and explained below:

1. **Handshake**: The hitchhiker keeps broadcasting requests to a potential driver until a threshold hitchhike wait time $T_{\mathrm{hwait}}$. Once a (potential) driver responds, the two robots exchange information. The hitchhiker request comprises of $\left\{R_{h},G_{h},P_{h}\right\}$, where, $R_{h}$ is the ID, $G_{h}$ is the goal location, and $P_{h}$ is the profile of the hitchhiking robot. The potential driver robot checks if the length of the common path ($d_{\mathrm{hh}}$) traversed during hitchhiking is longer than a threshold distance ($T_{\mathrm{dhh}}$). This is graphically shown in Figure 3, in which, $S_{d}$ and $S_{h}$ are the start locations, and $G_{d}$ and $G_{h}$ are the goal locations of the driver and hitchhiker robots, respectively. The common path between the points A and B is the hitchhiking distance ($d_{\mathrm{hh}}$). Hitchhiking is allowed if $d_{\mathrm{hh}}\geq T_{\mathrm{dhh}}$. Hitchhiking is denied for shorter distance (less than $T_{\mathrm{dhh}}$) due to the overhead involved in coupling and decoupling. Moreover, hitchhiking over shorter distances affects the service time. The threshold hitchhiking length ($T_{\mathrm{dhh}}$) depends on many factors like the dynamics of the environment, and the characteristics of the SLAM algorithm employed. A typical setting involves setting $T_{\mathrm{dhh}}$ to several meters (ex. ≈20 m). Notice that, from Figure 3, if $d_{\mathrm{hh}}\geq T_{\mathrm{dhh}}$, hitchhking is allowed even if the nodes $G_{d}$ and $G_{h}$ are far from each other. The best case scenario for hitchhking is when $G_{d}$ and $G_{h}$ completely overlap. This entire process is called a handshake. If no potential driver is found until $T_{\mathrm{hwait}}$ time, the hitchhiker navigates towards the goal on its own. A driver with high task priority ($T_{d}$) will simply ignore any requests from a hitchhiker.
2. **Coupling**: The next step is coupling between the hitchhiker and the driver. Coupling is defined as the process in which the hitchhiker aligns behind the driver robot and the QR-code behind the driver is recognized to initiate visual servoing. The alignment and coupling are only allowed within a threshold time $T_{\mathrm{align}}$ and $T_{\mathrm{coupling}}$, respectively. In order to assist coupling, the environment is marked with special pre-defined markers known to all the robots. Certain positions with markers are also reserved to further assist coupling.
3. **Navigation**: Once the robots are coupled, the driver initiates navigation towards the goal. During this time, the hitchhiker shut downs all the processes except visual servoing. In other words, the hitchhiker shuts down the localization, path planning, obstacle avoidance, and map update modules. It simply follows the driver robot using visual servoing. The driver robot executes all the modules.
4. **Decoupling**: Once the two robots have reached the destination, the decoupling process is initiated where visual servoing stops. During decoupling, the driver gives the current position location (i.e., the estimated x,y,θ pose in the map) and the uncertainty associated with it (Σ). This information must be given to the hitchhiker as it requires it as an initial estimate to localize itself in the map to navigate to another location. Moreover, during navigation if the driver robot has updated its map with the location of the new static obstacles (Ω), this information is also transferred to the hitchhiker to update its local map. This ensures that there is no loss of information during navigation for the hitchhiker.

The hitchhiker can thus skip redundant computation from the hitchhiking point to the decoupling location without any information loss.

## 3. Hitchhiking Points

Although hitchhiking can be initiated at any place in the map, it is generally not a good idea to do so because of the following two reasons:

1. Loss of Efficiency: Hitchhiking consumes time in alignment and coupling. By allowing hitchhiking anywhere in the map, the robots may not find fixed markers in the environment to assist coupling. In the absence of such markers, coupling is difficult and the robots consume more time. Moreover, the robots must always be alert of an incoming request from a hitchhiker.
2. Problem of Obstacles: Since it consumes time for the two robots to align and couple, hitchhiking at an inappropriate place is a hindrance in the pathways for other robots and people.

**Figure 4:**
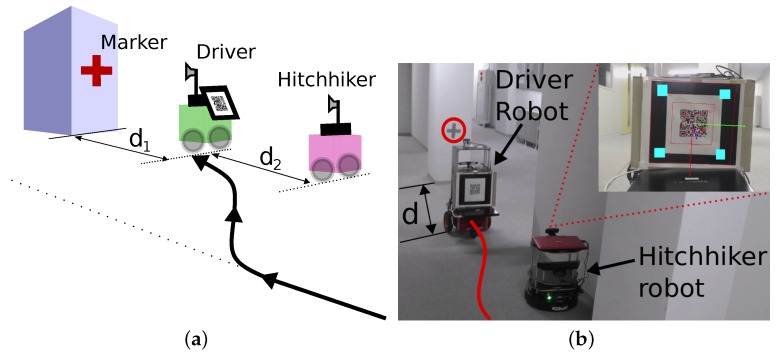
Benefit of hitchhiking area with markers. (**a**) Hitchhiker is stationary while the driver aligns with the marker; (**b**) Implementation result in real environment.

Therefore, we propose certain fixed ‘hitchhiking points’ in the map which are laid with artificial markers to assist coupling. It eliminates the aforementioned two problems and gives an estimate to the robots about a possibility of hitchhiking. Its major benefit is that the driver robot knows exactly where to stop, and trajectories can be generated beforehand for alignment. As shown in Figure 4a, the hitchhiker stands pre-aligned broadcasting requests, while the driver aligns with the marker at a certain distance ($d_{1}$). This arrangement automatically helps in the alignment process for coupling. Figure 4b shows the real example of such alignment and coupling at hitchhiking point.

### 3.1. When to Hitchhike and When Not

The best possible scenario for hitchhiking is when the start and goal locations of the hitchhiker and driver are the same. Hitchhiking is good for different goals if the common path distance is large for navigation.

In case the goal locations of the two robots are different, the point of decoupling in the map can easily be calculated. Our implementation uses A* [22] which is a famous algorithm for path planning. Let G=(V,E) is a graph with non-negative edge distances, and h is an admissible heuristic. Let $S_{\mathrm{hp}}$ be the hitchhiking point which marks the start location and $T_{h}$ be the end node of the hitchhiker robot. If d(v) is the shortest distance from $S_{\mathrm{hp}}$ to v seen so far, then d(v)+h(v) gives an estimate of the distance from $S_{\mathrm{hp}}$ to v, and similarly from v to $T_{h}$. The queue of nodes $Q_{h}=\left(V_{1},V_{2},\cdots ,V_{n}\right)$ sorted by d(v)+h(v) is the A* path from $S_{\mathrm{hp}}$ to $T_{h}$. Similarly, if $Q_{d}$ is the sorted node queue of the hitchhiker robot to $T_{d}$, then the farthest node in $Q_{d}\cap Q_{h}$ is the node of decoupling in the map.

However, hitchhiking is not good for short distances due to the extra time required for alignment, coupling, and decoupling which may adversely affect the service response time. Moreover, a hitchhiker always hitchhikes a robot with better or same profile score ($P_{\mathrm{id}}$) checked during the handshake step. This is to ensure that, ‘*you don’t trust suspicious drivers*’. This is natural as a robot with a robust and accurate SLAM module should never hitchhike another robot with less accurate sensors and SLAM module as doing so has risks of task failure for both the robots. On the other hand, a hitchhiker with a lower profile can always benefit from a driver with good profile in terms of accuracy of maps, navigation, obstacle avoidance, and localization.

## 4. Problem of ‘Driver Lost’ Scenario

One potential problem in hitchhiking is that during navigation the driver might be ‘lost’. This particularly depends on the robustness of the visual servoing algorithm employed. Visual servoing, particularly image based visual servoing is not very robust to large rotations. A detailed explanation of problems in visual servoing can be found summarized in the work of C. Francois [23] and can result in a driver lost scenario in which the follower robot is left behind while the driver robot navigates to its goal.

**Figure 5:**
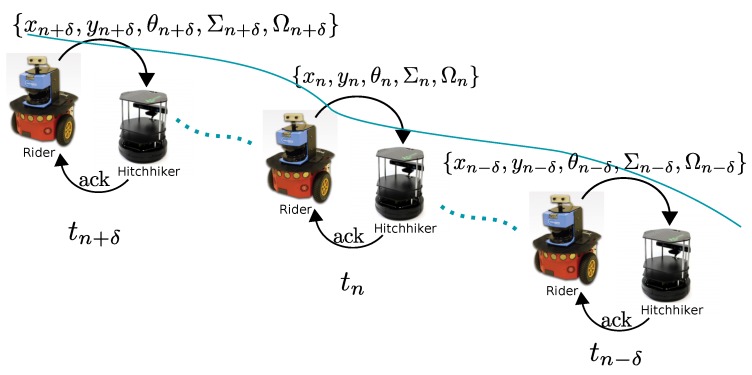
Intermittent information transfer from driver every δ time-steps to recover from ‘driver-lost’ scenario.

Since the hitchhiker has just visual servoing module in execution, if the driver is lost then it is difficult for the hitchhiker to localize itself in the map as it is completely unaware of its current position in the map. This problem is similar to the famous ‘*kidnapped robot problem*’ for which solutions are available in literature [24,25,26]. However, we propose to recover from this problem in the first place by transferring the current estimated pose ($x_{\delta },y_{\delta },\theta_{\delta }$) and associated uncertainty ($\Sigma_{\delta }$) information to the hitchhiker intermittently in intervals of δ s. This is graphically shown in Figure 5 where a driver is shown transferring information intermittently. With this scheme, even if the driver robot is lost due to failure of visual servoing, the hitchhiker still has a rough initial estimate to localize itself in the map, and navigate towards the goal independently.

As shown in Figure 5, the hitchhiker acknowledges the receipt of the intermittent information ($x_{\delta },y_{\delta },\theta_{\delta }$). Through this acknowledgement message (ack), the driver robot can know that the hitchhiker is following well and continue its navigation. In the absence of ack message, the driver stops for the hitchhiker to catch-up. A straightforward pseudo-code for hitchhiking is given in Algorithms 1 and 2 for driver and hitchhiker robots, respectively.

**Algorithm 1:** Hitchhiking Pseudocode (Driver Robot)
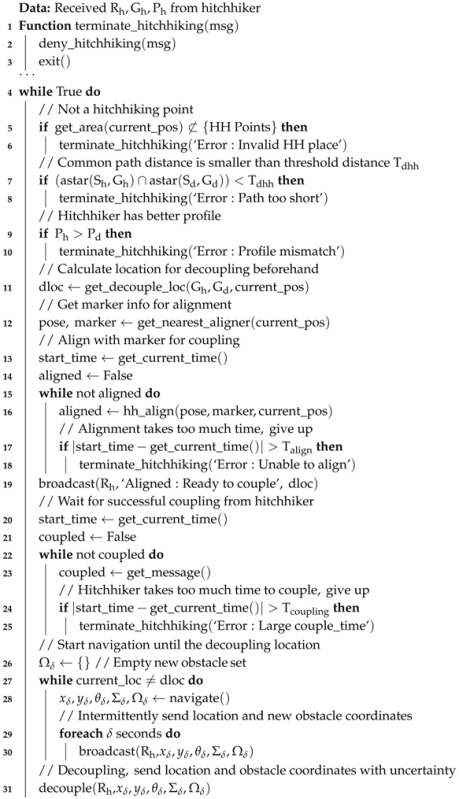


**Algorithm 2:** Hitchhiking Pseudocode (Hitchhiker)
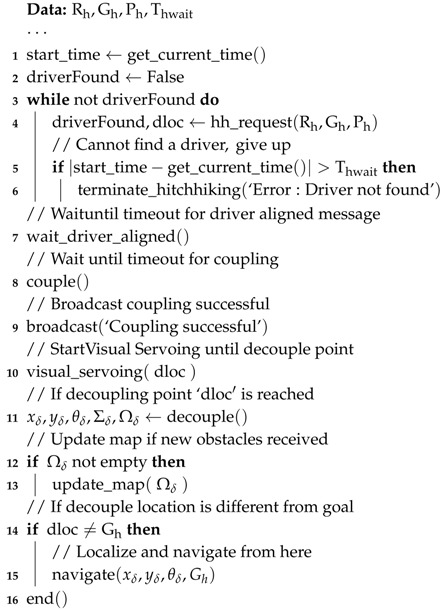


## 5. Award Mechanism

An award mechanism is designed in which the driver robot is awarded with points for assisting other robots in hitchhiking. The points are directly proportional to the distance traveled, i.e., Points=κ·f(hhike_start,hhike_end), where κ is a constant and the function f(·) calculates the distance between the start and end locations of hitchhiking. Robots try to increase their points whenever possible. Two constraints check that the task performance is not compromised: (a) Hitchhiking is not allowed over short distances; (b) Hitchhiking is only allowed when the driver robot has a better or same profile, and either robot has no time-critical task at hand.

## 6. State of the Art in Robot Localization

In this section, we discuss the state of the art related to robot localization and mapping in indoor environments. It is important for the driver and the hitchhiker robots to localize themselves in the map to ascertain the hitchhiking points, and the map needs to be updated by the driver robot. SLAM is a challenging problem due to the uncertainties of sensors and robot motion [27]. Bayes filters [28] are the most common tools to mathematically model these uncertainties. There are many variants of Bayes filter like Extended Kalman Filter (EKF), Unscented Kalman Filter (UKF), and Particle Filter (PF).

An Unscented Kalman Filter (UKF) performs a stochastic linearization through statistical linear regression process. The main idea behind UKF is that it is easier to approximate the probability function than the nonlinear function. A detailed explanation of UKF can be found in [29,30,31,32,33,34,35].

Particle Filter (PF) is a non-linear state estimator based on Bayesian filtering. It is a sequential Monte Carlo based technique which models the probability density using a set of discrete points. PF can represent a much broader space of distributions than, for example, Gaussians. A detailed explanation of Particle Filter can be found in [31,33,35,36,37].

Apart from the above mentioned filters, there are many other filters like histogram filter, information filter, and others which have been explained in detail in [31,33,34]. All of these filters can estimate the state of the robot and the uncertainty associated with the estimate. Readers may find a comparison of the merits and limitations of various filters in [38].

Recently, several fusion filters which are more robust have also been proposed. Work in [39] proposes a Fusion-Kalman/UFIR Filter. It is a fusion of Kalman Filter which is optimal but not robust, with the unbiased finite-impulse response (UFIR) filter which is more robust than KF but not optimal. The fusion is achieved by employing KF and UFIR as subfilters which provides small errors at the point where UFIR meets Kalman by applying probabilistic weights to each subfilter. A novel Deadbeat Dissipative FIR Filter with a finite impulse response (FIR) structure for linear discrete-time systems with external disturbance is proposed in [40] which ensures (Q,S,R)-α-dissipativity based on three slack matrix variables. A Hybrid-Particle/FIR Filter to improve the reliability of Particle Filter based localization has been proposed in [41,42] which detects PF failure and recovers localization by resetting the PF using the output of an auxiliary FIR filter. This makes PF which traditionally suffers from the sample impoverishment in noisy environments to be more reliable. Particularly, graph based SLAM algorithms [43], in which, a graph whose nodes correspond to the poses of the robot at different points in time and whose edges represent constraints between the poses, have been shown to be very successful. A detailed survey of SLAM techniques in the past 30 years, considering future challenges can be found in [44].

Notice that, the proposed hitchhiking in multi-robot systems is not limited to any one particular localization approach. In fact, any of the mapping and localization methods can be used with the robots. Obviously, the more robust and accurate the localization algorithm employed, more accurate is the navigation. The choice of the SLAM algorithm used may depend on factors like the sensors and computation devices available. Although any of the filters can be used, for the sake of completeness, we briefly discuss robot localization with Extended Kalman Filter (EKF).

### 6.1. Driver Robot Localization with Extended Kalman Filter

EKF based SLAM and localization has extensively been employed with mobile robots. Other variants of EKF like particle filters, and unscented kalman filter (UKF) are also very popular. EKF is a powerful mathematical tool to model the uncertainties of the sensors attached to the robot, and has been demonstrated successfully with laser range sensors, vision sensors, and intertial sensors, etc. An elaborated description of EKF can be found in [31].

The state of the robot ($x_{t}$) at time *t* is indicated by a vector comprising of its pose ${\left[x\;\;y\right]}^{T}$ and orientation (θ) as, $x_{t}={\left[x\;\;y\;\;\theta \right]}^{T}$. EKF assumes a Gaussian distribution in which the belief $bel(x_{t})$ at time *t* is given by the mean $\mu_{t}$ and the covariance $\Sigma_{t}$. At the start of navigation, the robot assumes no uncertainty, and $\Sigma_{t=0}$ is set to a zero matrix. The robot is made to move by issuing a command which comprises of the translation velocity ($v_{t}$) and rotational velocity ($\omega_{t}$) as ${\left[v_{t}\;\;\omega_{t}\right]}^{T}$.

(3) $$\begin{array}{c} \theta \leftarrow \mu_{t-1,\theta } \end{array}$$

To handle the non-linearity of the system, EKF uses Jacobians of motion and control functions. The Jacobian of motion function with respect to state is given by,

(4) $$\begin{array}{c} G_{t}\leftarrow \left[\begin{array}{ccc} 1 & \;\;\;\;0 & \;\;\;\;-\frac{v_{t}}{\omega_{t}}\cos\;\theta +\frac{v_{t}}{\omega_{t}}\cos\left(\theta +\omega_{t}\Delta t\right)\\ 0 & \;\;\;\;1 & \;\;\;\;-\frac{v_{t}}{\omega_{t}}\sin\;\theta +\frac{v_{t}}{\omega_{t}}\sin\left(\theta +\omega_{t}\Delta t\right)\\ 0 & \;\;\;\;0 & \;\;\;\;1 \end{array}\right], \end{array}$$

and the Jacobian of motion with respect to control is given by,

(5) $$\begin{array}{c} V_{t}=\left[\begin{array}{cc} \frac{-\sin\;\theta +sin(\theta +\omega_{t}\Delta t)}{\omega_{t}} & \;\;\;\frac{v_{t}\left(\sin\;\theta -\sin\left(\theta +\omega_{t}\Delta t\right)\right)}{\omega_{t}^{2}}+\frac{v_{t}\left(\cos\left(\theta +\omega_{t}\Delta t\right)\Delta t\right)}{\omega_{t}}\\ \frac{\cos\;\theta -cos(\theta +\omega_{t}\Delta t)}{\omega_{t}} & -\frac{v_{t}\left(\cos\;\theta -\cos\left(\theta +\omega_{t}\Delta t\right)\right)}{\omega_{t}^{2}}+\frac{v_{t}\left(\sin\left(\theta +\omega_{t}\Delta t\right)\Delta t\right)}{\omega_{t}}\\ 0 & \Delta t \end{array}\right]. \end{array}$$

With robot specific error-parameters $\alpha_{1},\cdots ,\alpha_{4}$, the covariance of noise in control space is given by,

(6) $$\begin{array}{c} M_{t}=\left[\begin{array}{cc} \alpha_{1}v_{t}^{2}+\alpha_{2}\omega_{t}^{2} & 0\\ 0 & \alpha_{3}v_{t}^{2}+\alpha_{4}\omega_{t}^{2} \end{array}\right]. \end{array}$$

Here, $\alpha_{1},\cdots ,\;\alpha_{4}$ are robot specific parameters. They are determined empirically and vary from robot to robot [31]. The prediction updates in state (${\bar{\mu }}_{t}$) and covariance (${\bar{\Sigma }}_{t}$) are given by,

(7) $$\begin{array}{c} {\bar{\mu }}_{t}=\mu_{t-1}+\left[\begin{array}{c} \frac{-v_{t}}{\omega_{t}}\sin\;\theta +\frac{v_{t}}{\omega_{t}}\sin\left(\theta +\omega_{t}\Delta t\right)\\ \frac{v_{t}}{\omega_{t}}\cos\;\theta -\frac{v_{t}}{\omega_{t}}\cos\left(\theta +\omega_{t}\Delta t\right)\\ \omega_{t}\Delta t \end{array}\right], \end{array}$$

and,

(8) $$\begin{array}{c} {\bar{\Sigma }}_{t}=G_{t}\Sigma_{t-1}G_{t}+V_{t}M_{t}V_{t}^{T}, \end{array}$$

respectively. The mapping from motion noise in control space to motion noise in state space is provided by the term $V_{t}M_{t}V_{t}^{T}$ in Equation (8).

To model the correction step, we assume that the sensors provide the range ($r_{t}$), bearing ($\phi_{t}$), and signature ($s_{t}$, for ex. color) of the landmark relative to the robot’s current pose ($x_{t}$). The covariance ($Q_{t}$) of the sensor noise is given by the matrix,

(9) $$\begin{array}{c} Q_{t}=\left[\begin{array}{ccc} \sigma_{r}^{2} & 0 & 0\\ 0 & \sigma_{\phi }^{2} & 0\\ 0 & 0 & \sigma_{s}^{2} \end{array}\right]. \end{array}$$

Let ${\left[m_{ix}\;\;m_{iy}\right]}^{T}$ be the coordinates of the *i*th landmark obtained by measurement $z_{t}^{i}={\left[r_{t}^{i}\phi_{t}^{i}s_{t}^{i}\right]}^{T}$ from the current pose ${\bar{\mu }}_{t}$, and *q* represent the squared distance as,

(10) $$\begin{array}{c} q={\left(m_{k,x}-{\bar{\mu }}_{t,x}\right)}^{2}+{\left(m_{k,y}-{\bar{\mu }}_{t,y}\right)}^{2}, \end{array}$$

then, we have,

(11) $$\begin{array}{c} {{\hat{z}}_{t}}^{k}=\left[\begin{array}{c} \sqrt{q}\\ atan2\left(m_{k,y}-{\bar{\mu }}_{t,y},m_{k,x}-{\bar{\mu }}_{t,x}\right)-{\bar{\mu }}_{t,\theta }\\ m_{k,s} \end{array}\right]. \end{array}$$

The Jacobian of measurement with respect to state is given by,

(12) $$\begin{array}{c} H_{t}^{k}=\left[\begin{array}{ccc} -\frac{m_{k,x}-{\bar{\mu }}_{t,x}}{\sqrt{q}} & -\frac{m_{k,y}-{\bar{\mu }}_{t,y}}{\sqrt{q}} & 0\\ \frac{m_{k,y}-{\bar{\mu }}_{t,y}}{q} & -\frac{m_{k,x}-{\bar{\mu }}_{t,x}}{q} & -1\\ 0 & 0 & 0 \end{array}\right] \end{array}$$

This gives the measurement covariance matrix as,

(13) $$\begin{array}{c} S_{t}^{k}=H_{t}^{k}\bar{\Sigma_{t}}{\left[H_{t}^{k}\right]}^{T}+Q_{t}. \end{array}$$

Maximum likelihood estimate is applied for all the *k* landmarks (Equations (10)–(13)) in the map to calculate the most likey correspondence j(i) as,

(14) $$\begin{array}{c} j\left(i\right)=\mathrm{argmax}\;\;\frac{1}{\sqrt{\det(2\pi S_{t}^{k})}}\;e^{-\frac{1}{2}{\left(z_{t}^{i}-{\hat{z}}_{t}^{k}\right)}^{T}{\left[S_{t}^{k}\right]}^{-1}\left(z_{t}^{i}-{\hat{z}}_{t}^{k}\right)}. \end{array}$$

The calculation of Kalman gain ($K_{t}$) and EKF updates for state ($\mu_{t}$) and covariance ($\Sigma_{t}$) only corresponds to this most likely estimate,

(15) $$\begin{array}{cc} K_{t}^{i} & ={\bar{\Sigma }}_{t}{\left[H_{t}^{j(i)}\right]}^{T}{\left[S_{t}^{j(i)}\right]}^{-1}\\ \mu_{t} & ={\bar{\mu }}_{t}+K_{t}^{i}\left(z_{t}^{i}-{\hat{z}}_{t}^{j(i)}\right)\\ \Sigma_{t} & =\left(I-K_{t}^{i}H_{t}^{j(i)}\right){\bar{\Sigma }}_{t} \end{array}$$

Thus, at each time step (*t*), a Kalman gain ($K_{t}$) is calculated from which the state ($\mu_{t}$) and covariance ($\Sigma_{t}$) are updated by the robot. In traditional navigation schemes, each robot of the multi-robot system must execute localization using the above mentioned computationally expensive steps. However, in the presented scheme only the driver robot executes localization while the hitchhiker follows the driver using visual servoing.

### 6.2. Pose Transfer during Decoupling

During decoupling the driver transfers its pose ($P_{d}={\left[x_{d}\;\;y_{d}\;\;\theta_{d}\right]}^{T}$) to the hitchhiker robot and the uncertainty associated with it ($\Sigma_{d}$). It is required as an initial estimate for the hitchhiker robot to localize itself in the map to navigate to another location.

Since the hitchhiker follows the driver using the QR-code and camera setup, the final orientation of the hitchhiker ($\theta_{h}$) is same as that of the driver robot, i.e.,

(16) $$\begin{array}{c} \theta_{h}=\theta_{d}. \end{array}$$

**Figure 6:**
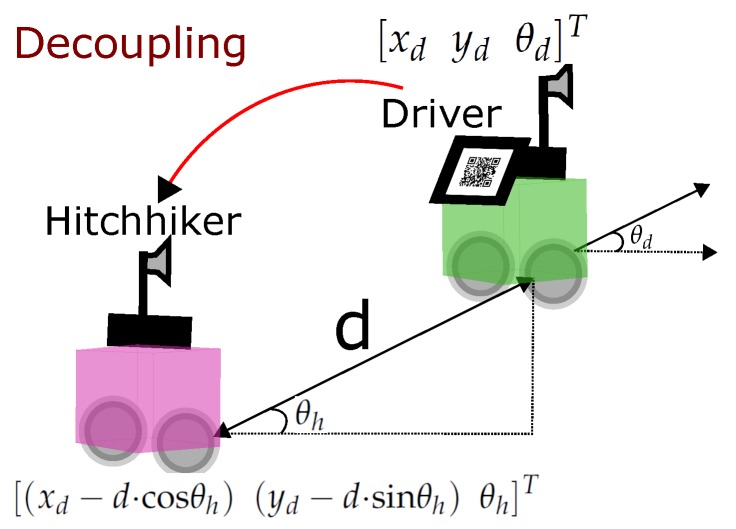
Decoupling process.

If *d* is the distance between the hitchhiker and the driver during decoupling, then the pose of hitchhiker is calculated as (Figure 6),

(17) $$\begin{array}{c} P_{h}={\left[\;\;\left(x_{d}-d\;\cdot \;\cos\;\theta_{h}\right)\;\;\;\;\;\;\left(y_{d}-d\;\cdot \;\sin\;\theta_{h}\right)\;\;\;\;\;\;\theta_{d}\;\;\right]}^{T}. \end{array}$$

Moreover, the hitchhiker assumes the same uncertainty in its pose as the driver robot, i.e.,

(18) $$\Sigma_{h}=\Sigma_{d}.$$

The hitchhiker robot uses this pose ($P_{h}$) to localize itself in the map. It can use the uncertainty ($\Sigma_{h}$) information to consider the distribution of particles (for ex. in case of particle filter [31,33,36,37]) by taking the Eigenvalue-Eigenvector decomposition of $\Sigma_{h}$. Eigenvalues ($\lambda_{1},\cdots ,\;\lambda_{n}$) and eigenvectors ($\vec{v_{1}},\cdots ,\;\vec{v_{n}}$) of the matrix $\Sigma_{h}$ gives the magnitude and direction of variance, respectively for considerable distribution of particle poses.

## 7. Experimental Results

This section presents the results of the experiments. We first show the motion model of the robots used in the experiment, and then discuss the results in different cases of hitchhiking.

### 7.1. Motion Model

**Figure 7:**
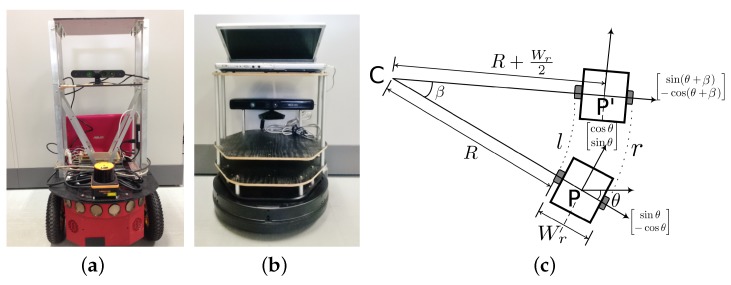
Robots used in the experiments. (**a**) Pioneer P3DX; (**b**) Kobuki Turtlebot; (**c**) Motion model of two wheel differential drive robots.

We used Pioneer-P3DX (Figure 7a) [45] and Kobuki Turtlebot (Figure 7b) [46] robot which are two wheeled differential drive robots [47]. Both the robots were equipped with distance sensors (Microsoft Kinect [48] and UHG-08LX laser range sensor [49]) and cameras. The distance sensor is accurate within ±30 mm within 1m, and within 3% of the detected distance between 1 and 8 m. The angular resolution is approx 0.36 degrees, and other specifications can be found in [49]. We first describe the motion model of the robot. The distance between the left and the right wheel is $W_{r}$, and the robot state at position *P*, is given as [x,y,θ]. From Figure 7c, turning angle β is calculated as,

(19) $$\begin{array}{cc} r & =\beta \;\cdot \;(R+W_{r}),\;\;\;l=\beta \;\cdot \;R\\ ∴\beta & =\frac{r-l}{W_{r}} \end{array}$$

and the radius of turn *R* as,

(20) $$\begin{array}{c} R=\frac{l}{\beta },\;\;\;\beta \neq 0. \end{array}$$

The coordinates of the center of rotation (*C*, in Figure 7c), are calculated as,

(21) $$\left[\begin{array}{c} Cx\\ Cy \end{array}\right]=\left[\begin{array}{c} x\\ y \end{array}\right]-\left(R+\frac{W_{r}}{2}\right)\;\cdot \;\left[\begin{array}{c} \sin\;\theta \\ -\cos\;\theta \end{array}\right]$$

The new heading $\theta^{\prime }$ is,

(22) $$\theta^{\prime }=\left(\theta +\beta \right)\;\mathrm{mod}\;2\pi ,$$

from which the coordinates of the new position $P^{\prime }$ are calculated as,

(23) $$\left[\begin{array}{c} x^{\prime }\\ y^{\prime } \end{array}\right]=\left[\begin{array}{c} Cx\\ Cy \end{array}\right]-\left(R+\frac{W_{r}}{2}\right)\;\cdot \;\left[\begin{array}{c} \sin\;\theta^{\prime }\\ -\cos\;\theta^{\prime } \end{array}\right],\;\;\beta \neq 0\;\Rightarrow r\neq l.$$

If r=l, i.e., if the robot motion is straight, the state parameters are given as, $\theta^{\prime }=\theta$, and,

(24) $$\begin{array}{c} \left[\begin{array}{c} x^{\prime }\\ y^{\prime } \end{array}\right]=\left[\begin{array}{c} x\\ y \end{array}\right]+l\;\cdot \;\left[\begin{array}{c} \cos\;\theta \\ \sin\;\theta \end{array}\right],\;\left(l=r\right). \end{array}$$

**Figure 8:**
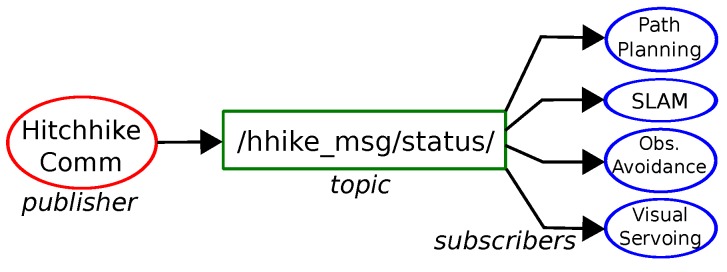
Simplified view of ROS topic (green), publisher (red) and subscriber (blue) nodes for module on/off.

Different experiments in real indoor environment were performed to test the proposed hitchhiking in various scenarios. Pioneer P3DX was the driver robot, and Turtlebot initated hitchhiking in all the cases. Both the robots used ROS [50] on Ubuntu computer and were on the same network to communicate with each other. Figure 8 shows a simplified view of ROS topics and nodes. The hitchhike communication module publishes an appropriate message on a topic which is subscribed by the other modules to turn them on or off.

For visual servoing, we used a modified open source Visp (Visual Servoing Platform) library [20,51,52] for online tracking. The initial pose was set by extracting the location of the four QR-code corners using a Perspective-n-Point (PnP) algorithm [53] from which a model based tracker was initialized to extract the black area around the QR-code. A hybrid approach for tracking edges and keypoint features was employed to estimate the pose. The robots exchanged messages in JSON format for which JSMN parser was used [54]. A sample JSON message is given in Appendix A: Listing 1.

### 7.2. Experiments in Which Hitchhiking Was Allowed

Three sets of the following two experiments were conducted with permissible initial conditions of hitchhiking. For comparison, the two experiments were performed with and without the new obstacles in the environment.

#### 7.2.1. Experiment 1

**Figure 9:**
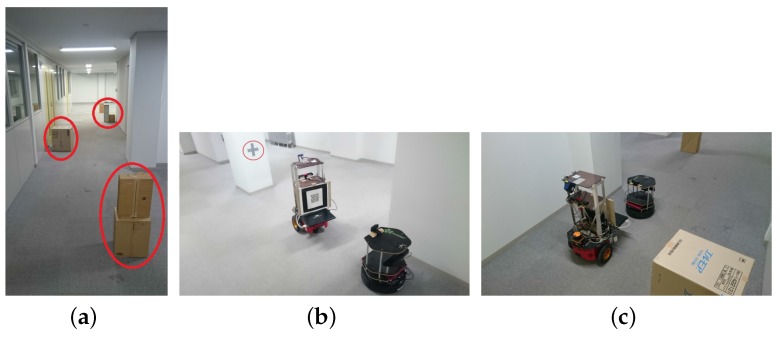
Experiment 1. (**a**) Environment with new obstacles marked in red; (**b**) Coupling between the two robots. The ‘+’ marker on pillar is marked in red circle; (**c**) Turtlebot following P3DX through visual servoing. (Refer the accompanied video provided in the Supplementary Materials section).

Experiment 1 was carried out in the corridor (≈23 m long, 2.23 m wide) shown in Figure 9a. In order to test map update and new obstacle coordinate transfer, the boxes marked in red circles in Figure 9a were placed as new obstacles and were not reflected in the old maps of the two robots. Turtlebot waited and initiated the hitchhiking process. The hitchhiking point with a ‘+’ sign marked on the pillar is shown in Figure 9b. Driver P3DX robot aligned itself with the marker on the pillar whereas the Turtlebot stood pre-aligned for visual servoing. The navigation started and Turtlebot followed the driver P3DX as shown in Figure 9c. The hitchhiker Turtlebot had temporarily shut down its modules except visual servoing.

**Figure 10:**
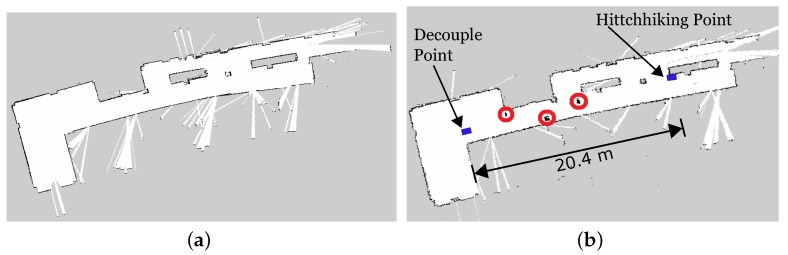
Experiment 1. (**a**) Initial map without obstacles; (**b**) Updated map with new obstacles. Driver robot transferred the obstacle coordinates (marked in red) to the hitchhiker.

Figure 10 shows the results of the map update process of the experiment. Figure 10a shows part of the map without the obstacles. Both Turtlebot and P3DX had this map before starting the experiment. Figure 10b shows the updated map with the positions of the new obstacles marked in red circles. The driver successfully transferred the coordinates of the new obstacles to the hitchhiker during the decoupling process. The actual values of the coordinates are summarized in Table 1. For the sake of simplicity, both the robots in our implementation had the prior grid maps build from the same location giving them the same anchor which simplified the process of transferring the new obstacle information. In case of map builds from different locations, the relevant transformation can be calculated for 2D [55,56] and 3D [57] cases. The dimensions of the obstacles in Table 1 is in grid pixels.

#### 7.2.2. Experiment 2

**Figure 11:**
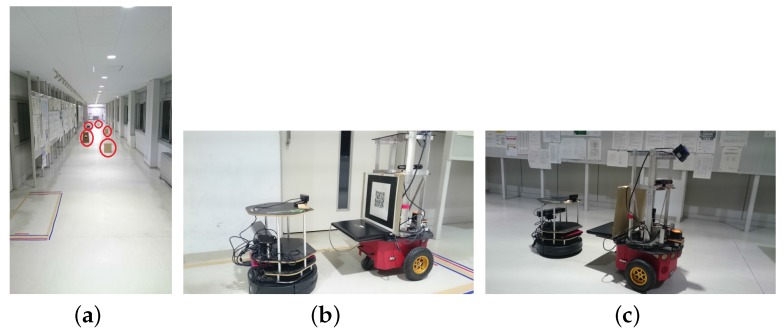
Experiment 2. (**a**) Environment with new obstacles marked in red; (**b**) Coupling between the two robots using left wall as marker (**c**) Turtlebot following P3DX through visual servoing. (Refer the accompanied video provided in the Supplementary Materials section).

Experiment 2 was carried in a long corridor (≈30 m long, 2.92 m wide) shown in Figure 11a. Similar to the previous experiment, the boxes marked in Figure 11a were placed as new obstacles. The hitchhiking Turtlebot was not pre-aligned and there were no artificial markers to assist coupling. Instead, the left wall was used as a landmark. The coupling process is shown in Figure 11b. The navigation started and Turtlebot followed the driver P3DX as shown in Figure 11c.

**Figure 12:**
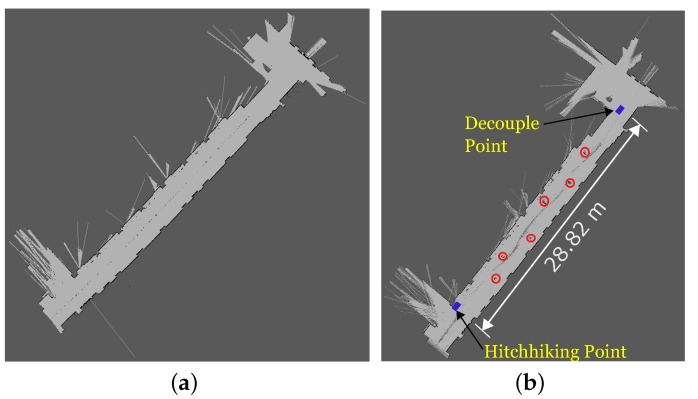
Experiment 2. (**a**) Initial map without obstacles; (**b**) Updated map with new obstacles. Driver robot transferred the obstacle coordinates (marked in red) to the hitchhiker.

Figure 12 shows the results of the map update process of the experiment. Figure 12a was the section map without the obstacles. Figure 12b shows the updated map with the positions of the new obstacles marked. The driver successfully transferred the coordinates of the new obstacles to the hitchhiker during the decoupling process which has been summarized in Table 1. The accuracy of obstacle locations were verified manually in both the experiments.

**Figure 13:**
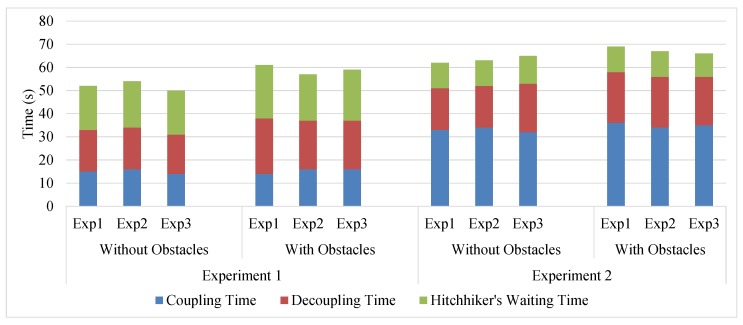
Coupling, decoupling, and hitchhiker’s waiting time in different experiments done with/without new obstacles.

The time taken in coupling, decoupling, and waiting for the driver has been summarized in Figure 13 for the three runs of the two experiments with and without new obstacles. Table 2 shows the average time of the three runs. The total path distance navigated in Experiments 1 and 2 were, 20.4 m and 28.82 m, respectively. To award 10 points to the driver for every 25 m hitchhike, κ (Section 5) was set to 0.4, and the driver robot was awarded 8.2 and 11.5 points, respectively.

### 7.3. Experiments with Denied Hitchhiking

We performed three more experiments to test scenarios in which hitchhiking should be denied. Experiments were performed in the same setup (Figure 11a) as in experiment 2. (a) In the first case, we set the priority of the driver robot to a high value of 10 (the lowest value being 1 in our implementation). Due to the high task priority, the driver P3DX ignored the requests from the hitchhiker; (b) In the second case, we reversed the profile scores of the two robots i.e., we set the driver P3DX with a profile score of 58, and hitchhiker with a profile score of 90. In this case, the driver stopped upon receiving the request from the hitchhiker, however, the hitchhiking was denied as the the driver had a lesser profile score. Notice that in this case, the two robots can reverse the roles, i.e., the requesting robot with better profile score can be the driver and other hitchhiker. However, we did not consider this case in the current work and will do so in future work; (c) The third case scenario was tested at a wrong hitchhiking location and the driver ignored the requests from the hitchhiker.

### 7.4. Transferring Quality Maps to Hitchhiker

**Figure 14:**
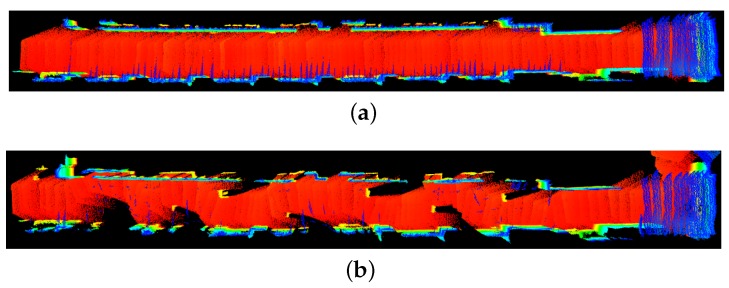
Top view of the 3D point cloud map in Experiment 2. (**a**) Initial map without obstacles; (**b**) Map with obstacles.

In both the previous experiments, apart from the occupancy grid map based map update, 3D map update was also performed by the driver robot. The initial point cloud map of the environment before placing new obstacles in experiment 2 is shown in Figure 14a. The final updated 3D point cloud map with the new obstacles is shown in Figure 14b.

**Figure 15:**
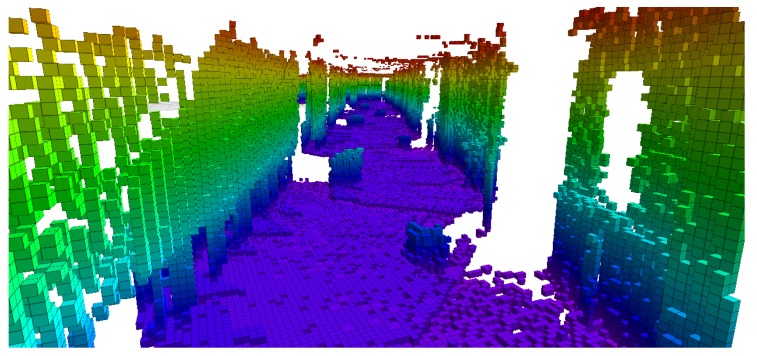
Octomap of the point cloud map shown in Figure 14b.

Notice that not only the locations of the new obstacles, but high quality 3D maps can also be transferred to the hitchhiker during decoupling. This is particularly useful if the hitchhiker’s profile is poor and it is not equipped with accurate 3D sensors or powerful computational units. In that case, a driver with good sensors and powerful computer can build high quality maps and give to the hitchhiker robot. Algorithms like OctoMap [58] can be used to get reduced sized maps from 3D point cloud maps, as shown in Figure 15.

## 8. Discussion

The results in Section 7 show that hitchhiking can be implemented in multi-robot systems with a simple setup. The hardware required are just QR-code marker, camera, and wireless communication setup. The software module can be implemented with a module on/off switch and visual servoing module. The measure of computation saved by the hitchhiker lies on many factors like the algorithms employed for path-planning, SLAM, and navigation. It also depends on the computation units, robot specifications, and the topology of the environment. Moreover, since the implementation and execution platforms vary and evolve rapidly, we focus on which redundant modules can be turned off without affecting the quality of service and loss of information for the hitchhiker. Moreover, compared to the other modules, SLAM particularly is a computationally expensive module and the hitchhiker can save substantial computation by skipping it. Table 3 shows the different modules run by the two robots in navigation in the experiments in Section 7. It is clear that the traditional navigation requires all the modules of both the robots to be active. On the other hand, in hitchhiking, most of the modules of the hitchhiker are off. One overhead is visual servoing which is not computationally expensive compared to SLAM (especially 3D SLAM). Another overhead is the delay in service time caused due to hitchhiking. Table 2 summarizes the average time required for coupling, decoupling, waiting for a potential driver, and the driver’s total delay. Although it depends upon the exact task, however, for less critical tasks, a delay of about 70 s should be acceptable.

It is interesting to notice in Table 2 that there is little time difference in the experiments with and without obstacles. The presence of obstacles only affect the actual navigation time. Moreover, in Experiment 2 the hitchhiker was not pre-aligned and there were no markers and therefore the average time for coupling in Experiment 2 was more than that in Experiment 1. This shows that having hitchhiking points with markers in the map can save coupling time. As shown in Section 7.3, hitchhiking is denied for high priority tasks. Moreover, as described in Section 2.2, the hitchhiker only waits for a potential driver for $T_{\mathrm{hwait}}$ time, which ensures that service is not affected adversely. Although our implementation dealt with local robot communication, hitchhiking can be very efficient with global communication in a sensor network, as the hitchhiker can know beforehand the passage of a potential driver with desired characteristics near a hitchhiking area. The proposed hitchhiking is not limited to a particular localization algorithm and any of the robust localization algorithms can be employed. Although the results were presented using EKF based localization algorithms, more robust algorithms (ex. [39,40,41,43]) will improve the accuracy of the results. Robustness of the visual servoing and communication modules largely determine the success of hitchhiking.

## 9. Summary

This paper introduced a novel idea of hitchhiking in multi-robot systems. When two robots have a common path towards the goal, the follower hitchhiker can skip the redundant computation of path planning, obstacle avoidance, map update, and localization. The process of hitchhiking was systematically explained in four steps. Later, the advantages of having hitchhiking points in the map were discussed. Solutions to the problem of ‘driver lost’ scenario were provided. The experimental results show that hitchhiking is feasible in a multi-robot system without any loss of information. Hitchhiking can particularly be useful in multi-robot systems in which some of the robots have less accurate sensors and less powerful computational resources. In future, we plan to test hitchhiking in dynamic environments. We also plan to test the role reversibility of robots based on their profiles.

## Figures and Tables

**Table 1:** New obstacle info transferred to the hitchhiker.

Obstacles	Experiment 1		Experiment 2		
	(x, y)	(Width × Breadth)	(x, y)	(Width × Breadth)
Obstacle 1	(231, 152)	(7×4)	(394, 685)	(11×11)
Obstacle 2	(308, 129)	(10×11)	(493, 650)	(14×11)
Obstacle 3	(376, 150)	(11×7)	(632, 698)	(10×12)
Obstacle 4	−	−	(795, 646)	(21×10)
Obstacle 5	−	−	(931, 682)	(17×11)
Obstacle 6	−	−	(1075, 654)	(15×10)

**Table 2:** Average time of the hitchhiking components.

Exp	Obstacles	Time to	Time to	Waiting Time	Delay of	Delay of
	Yes/No	Couple	Decouple	of Hitchhiker	Driver	Hitchhiker
Exp 1	No	15.00 s	17.67 s	19.33 s	32.67 s	52.00 s
	Yes	15.33 s	22.00 s	21.67 s	37.33 s	59.00 s
Exp 2	No	33.00 s	19.00 s	11.33 s	52.00 s	63.33 s
	Yes	35.00 s	21.67 s	10.67 s	56.67 s	67.33 s

**Table 3:** Modules run with and without hitchhiking.

Scheme	Robot	PP	OBS	LZN	MAP	COM	VS
Traditional	R1	On	On	On	On	On	Off
	R2	On	On	On	On	On	Off
Hitchhiking	R1	On	On	On	On	On	Off
	R2	Off	Off	Off	Off	On	On

PP: Path Planning, OBS: Obstacle Avoidance, LZN: Localization, MAP: Mapping, VS: Visual Servoing, COM: Communication.

## Supplementary Materials

Download link for video presentation with results: http://www.mdpi.com/1424-8220/17/8/1878/s1.

## Abbreviations

The following abbreviations are used in this manuscript:

$R_{h/d}$	Robot ID of hitchhiker (h)/driver (d)
$S_{h/d}$	Start location of hitchhiker (h)/driver (d)
$G_{h/d}$	Goal location of hitchhiker (h)/driver (d)
$T_{h/d}$	Task priority of hitchhiker (h)/driver (d)
$P_{h/d}$	Robot profile of hitchhiker (h)/driver (d)
$T_{\mathrm{hwait}}$	Threshold hitchhike wait time
$T_{\mathrm{align}}$	Threshold alignment time
$T_{\mathrm{coupling}}$	Threshold coupling time
$T_{\mathrm{dhh}}$	Threshold hitchhiking distance
Σ	Positional uncertainty of robot
Ω	New static obstacles
${\left[x,y,\theta \right]}^{T}$	Robot Pose
SLAM	Simultaneous Localization and Mapping
EKF	Extended Kalman Filter

## Appendix A

Listing 1: Example of driver message in JSON format.
